# Kinematic parameters after tibial nonunion treatment using the Ilizarov method

**DOI:** 10.1186/s12891-022-05683-1

**Published:** 2022-07-28

**Authors:** Łukasz Pawik, Felicja Fink-Lwow, Andżelika Pajchert Kozłowska, Łukasz Szelerski, Radosław Górski, Malwina Pawik, Paweł Reichert, Piotr Morasiewicz

**Affiliations:** 1 Department of Physiotherapy in Motor Disorders and Dysfunctions, Wroclaw University of Health and Sport Sciences, Al. IJ Paderewskiego 35, Wroclaw, Poland; 2 Department of Massage and Physical Therapy, Faculty of Physiotherapy, Wroclaw University of Health and Sport Sciences, Al. IJ Paderewskiego 35, Wroclaw, Poland; 3 Department and Clinic of Orthopaedic and Traumatologic Surgery, Wroclaw Medical University, Borowska 213, 50–556 Wroclaw, Poland; 4 Department of Orthopedics and Musculoskeletal Traumatology, Medical University of Warsaw, Lindeya 4, 02–005 Warsaw, Poland; 5 Department of Trauma and Hand Surgery, Wroclaw Medical University, Borowska 213, 50-556 Wroclaw, Poland; 6 Department of Orthopaedic and Traumatologic Surgery, Institute of Medical Sciences, University of Opole, al. Witosa 26, 45-401 Opole, Poland

**Keywords:** Gait, Kinematic parameters, Noraxon MyoMOTION System, Nonunion, Tibia, Ilizarov method

## Abstract

**Background:**

Analysis of the outcomes of Ilizarov treatment of tibial nonunion shows functional deficits in the lower limbs of some patients. Biomechanical gait parameters are an important measure for assessing musculoskeletal disorder treatments that aim to restore normal gait. The purpose of our study was to compare the kinematic parameters in patients with tibial nonunion treated using the Ilizarov method and those in a control group of healthy volunteers.

**Methods:**

The study population consisted of 23 patients (age 54.9 ± 16.4 years) who were treated for tibial nonunion using the Ilizarov method, as well as 22 healthy adult controls (age 52.7 ± 10.6 years). Kinematic parameters were measured using a Noraxon MyoMOTION System. We measured hip flexion and abduction, knee flexion, ankle dorsiflexion, inversion, and abduction during walking.

**Results:**

Our analysis showed significant differences between the patients’ operated limbs (OLs) and the controls’ nondominant limbs (NDLs) in the ranges of hip flexion, hip abduction, and knee flexion. We observed no significant differences in knee flexion between the OL and the NOL in patients or between the dominant limb (DL) and NDL in controls. Our evaluation of the kinematic parameters of the ankle joint demonstrated significant differences between the patients’ OLs and the controls’ NDLs in the ranges of ankle dorsiflexion, ankle inversion, and ankle abduction. There were also significant differences in the range of ankle dorsiflexion and ankle abduction between the patients’ NOLs and the controls’ DLs.

**Conclusion:**

Tibial nonunion treatment using the Ilizarov method does not ensure complete normalization of kinematic parameters assessed 24–48 months following the completion of treatment and rehabilitation.

## Introduction

The Ilizarov method is commonly used in the treatment of tibial nonunion [1–11]. However, analysis of treatment outcomes shows functional deficits in the lower limbs of some patients. As demonstrated elsewhere, these deficits may be a direct result of errors in the surgical technique itself or of imperfect fixation or stabilization [7–11]. Comorbidities, particularly metabolic disorders (such as type 2 diabetes mellitus), eating disorders, and modifiable lifestyle factors (such as smoking) may also play a role [12].

Biomechanical gait parameters are an important measure for assessing musculoskeletal disorder treatment aiming to restore normal gait. Restoring the physiological range of motion in the affected joints is thus the basis of optimal treatment [13–20].

There have been reports of patients with tibial nonunion who exhibited a limited range of motion, especially in the knee and ankle joints. There may be various reasons behind such dysfunction, including the exact nature of the original injury and complications of an earlier or most recent surgery [1, 3, 4, 6, 8, 9]. Accurate post-treatment range-of-motion assessment is important not only for patients, but also for orthopedic surgeons and physiotherapists. Identifying the range-of-motion abnormalities that require specific and more intense rehabilitation may improve final treatment outcomes.

The available literature seems to lack reports on kinematic parameter assessment following tibial nonunion treatment using the Ilizarov method. However, there have been a handful of studies assessing the range of motion in the ankle and knee joints at rest with goniometers in patients who had undergone osteotomy and used an Ilizarov external fixator [21–23]. At this point, we would like to stress that the use of goniometers guarantees neither high accuracy nor repeatability of measurements. The Noraxon MyoMOTION System, which we were the first to use in patients treated for tibial nonunion, can collect very accurate, repeatable, and objective range-of-motion data [24–27].

The purpose of our study was to compare selected kinematic parameters in patients with tibial nonunion treated using the Ilizarov method and in healthy volunteers.

## Methods

This retrospective study was approved by the local Bioethics Committee and conducted in accordance with the Code of Ethics of the World Medical Association (Declaration of Helsinki) for experiments in humans. The study was conducted between 2019 and 2020.

The study population consisted of 23 patients (seven females and sixteen males; age 54.9 ± 16.4 years*;* height 170.0 ± 11.0 cm; body weight 81.4 ± 14.0 kg; body mass index (BMI) 28.1 ± 3.9 kg/m^2^) who were treated for tibial nonunion using the Ilizarov method, as well as 22 healthy adult controls (ten females and twelve males; age 52.7 ± 10.6 years; height 172 ± 11.0 cm; body weight 75.9 ± 12.9 kg; BMI 26.3 ± 3.4 kg/m^2^) with no musculoskeletal dysfunction, referred by a physician or orthopedist. All subjects had given their informed consent to participate in the study.

The patients in our study had completed their treatment 24–48 months prior to the Noraxon MyoMOTION System measurements, and have completed their entire physiotherapy protocol, including a detailed and individually designed rehabilitation regimen.

The rehabilitation protocol was personalized based on each patient’s condition and his or her functional capacity. For four weeks following their operation, patients did active exercises of the hip, knee, and ankle joint of the OL while remaining within their pain tolerance, and isometric exercises (particularly for the vastus medialis oblique (VMO) muscle and the gluteus maximus and medius muscles). The rehabilitation regimen also covered facial therapy, proprioception exercises, and scar mobilization (starting from postoperative day 14). At the same time, the patients were taught how to walk with two elbow crutches on flat surfaces and stairs. Over the subsequent 4–6 weeks, exercises progressed in comparison to those from the earlier stage and were complemented with strengthening exercises for the NOL in the sitting position, balance exercises, manual therapy, and strengthening exercises with elastic bands. Subsequent stages of rehabilitation (postoperative weeks 8–10) focused on strengthening exercises in the standing position, balance exercises, and progression of the exercises from earlier stages, in order to optimally improve the range of motion and muscle strength.

Bone nonunion in the patient group had been a result of unsuccessful initial fracture treatment via intramedullary nail fixation (six cases) or plate fixation (seventeen cases). In all patients the Ilizarov method was the first treatment for tibial nonunion. Eighteen patients had hypertrophic tibial nonunion and five had atrophic tibial nonunion. The site of nonunion was the proximal, middle, and distal one-third of the tibial shaft in 2, 7, and 14 patients, respectively. In all patients the Ilizarov fixator was mounted only on the lower leg, without extending onto the thigh or foot, which left the knee and ankle mobile*.* After the treatment, none of the evaluated patients required limb lengthening, with limb shortening either absent or less than 1 cm, and none had permanent limb deformity in any plane. The mean duration of Ilizarov treatment was 185 days.

The range-of-motion values in the NOLs and OLs of patients were compared with those in the DLs and NDLs in the controls, respectively. The DL was identified by having the patient juggle or kick a ball [28, 29]. We also made a comparison between the OL and NOLs in the patients group.

Kinematic parameters were measured using a Noraxon (myoMuscle Master Edition System) MyoMOTION System (Scottsdale, AZ, USA), which comprises a set of 1–16 sensors using inertial sensor technology. Following a rigid-body model with sixteen joint segments used in the MR3 software, the Noraxon MyoMOTION System inertial sensors were placed on the foot (on the dorsal aspect, slightly below the malleoli), leg (on the anterior aspect, midway between the malleoli and the patella), on the thigh (the anterior aspect of the quadriceps femoris muscle, slightly above the patella), and in the sacral region. All sensors were attached by the same technician with the use of special straps and elastic adhesive tape. Each strap had a pocket for the inertial sensor. Calibration was carried out in the upright position in order to determine the value of the 0 angle in the joints. Sampling frequency for the inertial sensors was set at 200 Hz [24–27].

**Fig. 1:**
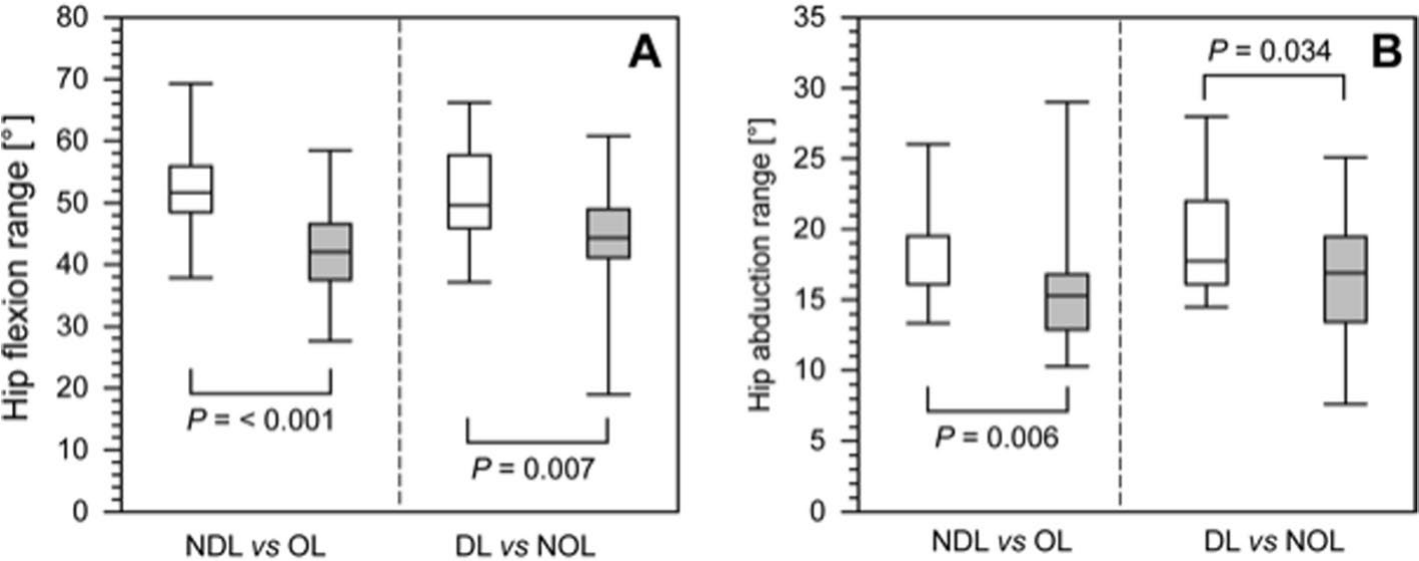
The comparison of hip flexion range (panel **A**) and hip abduction range (panel **B**) for nondominant limbs (NDLs) and operated limbs (OLs); dominant limbs (DLs), and nonoperated limbs (NOLs) between healthy controls and the patients after treatment with the Ilizarov method. The boundary of the box closest to zero indicates the 25^th^ percentile, the line within the box marks the median, and the boundary of the box farthest from zero indicates the 75^th^ percentile. The whiskers above and below the box indicate the 90th and 10th percentiles, respectively. White boxes, healthy people; filled boxes, patients

We measured hip flexion and abduction, knee flexion, and ankle dorsiflexion, inversion, and abduction during a twenty-meter walk along a straight line. Each person did at least four repetitions, and the mean values from at least two complete, correct walks were used in the analysis. The walk was performed in a straight line of at least 10 m. The first and the last two strides were excluded to avoid the acceleration and deceleration in gait. The walks were done barefoot, with no additional orthopedic aids, and were supervised by qualified orthopedists and physiotherapists (Fig. 1). The angle values were recorded with an accuracy of 0.1 and analyzed statistically. The maximum and minimum angle and range-of-motion values were calculated for the comparison of discrete variables during the gait cycle. The maximum (maximum range) and minimum (minimum range) joint angles and range of motion for the hip, knee, and ankle were calculated for all phases of gait. Positive values of the angle depending on the joint and axis correspond to: flexion, abduction, external rotation, dorsiflexion, and inversion.

### Statistical analysis

All analyses were conducted using the SigmaPlot v.13 statistics package (Systat Software, San Jose, California, USA). Continuous variables were first analyzed for a normal distribution using the Kolmogorov–Smirnov test with the Lilliefors correction. Data exhibiting a normal distribution were presented as means ± standard deviations (SDs), and an unpaired Student’s *t*-test was used to test the differences between the two groups. In the case of data that did not pass the normality test, the significance of differences was analyzed using the Mann–Whitney *U*-test, and the data were expressed as the medians and 5^th^ to 95^th^ percentile ranges. The level of statistical significance was set at *P* < 0.05.

## Results

**Fig. 2:**
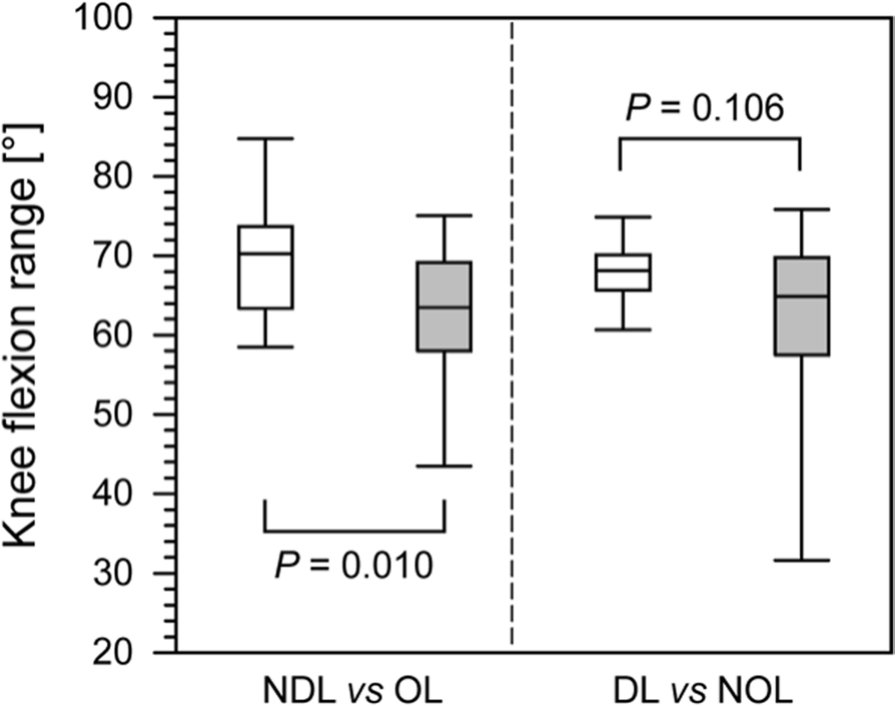
A comparison of the knee flexion range in the operated limbs (OLs) and non-operated limbs (NOLs) of patients after treatment with the Ilizarov method and the non-dominant limbs (NDLs) and dominant limbs (DLs) in the healthy group. The boundary of the box closest to zero indicates the 25^th^ percentile, the line within the box marks the median, and the boundary of the box farthest from zero indicates the 75^th^ percentile. The whiskers above and below the box indicate the 90^th^ and 10^th^ percentiles, respectively. White boxes, healthy people; filled boxes, patients

Our analysis showed significant differences between the OL in patients and the NDL in controls in the following parameters: the ranges of hip flexion (*P* < 0.001), hip abduction (*P* = 0.006), and knee flexion (*P* = 0.010) (Figs. 1 and 2). However, there were no significant differences in the knee flexion range between the patients’ NOLs and the controls’ DLs (*P* = 0.102) (Table 1).

**Table 1:** Differences in hip flexion, hip abduction, and knee flexion between patients who had undergone Ilizarov therapy and healthy controls

	Control group (*n* = 22)	Patients after surgery (*n* = 23)	*P*
Hip flexion min NDL^a^, OL^c^ [°]	-19.2 (-23.90 – -13.2)^a^	-12.4 (-23.80 – -4.0)^c^	** < 0.001**
Hip flexion min DL^b^, NOL^d^ [°]	-17.0 (-26.50 – -10.2)^b^	-13.3 (-19.70 – -2.8)^d^	**0.004**
*P*	0.244	0.911	
Hip flexion max NDL^a^, OL^c^ [°]	32.6 (22.6 – 47.2)^a^	29.7 (21.3 – 40.9)^c^	**0.016**
Hip flexion max DL^b^, NOL^d^ [°]	33.6 (19.5 – 50.8)^b^	31.6 (10.8 – 46.7)^d^	0.240
*P*	0.856	0.551	
Hip flexion range NDL^a^, OL^c^ [°]	51.6 (38.4 – 68.6)^a^	42.0 (29.0 – 57.9)^c^	** < 0.001**
Hip flexion range DL^b^,NOL^d^ [°]	49.6 (37.9 – 65.2)^b^	44.3 (20.9 – 60.5)^d^	**0.007**
*P*	0.419	0.632	
Hip abduction min NDL^a^,OL^c^ [°]	-9.8 (-17.5 – -1.2)^a^	-8.1 (-14.1 – -0.7)^c^	0.231
Hip abduction min DL^b^, NOL^d^ [°]	-6.9 (-16.5 – -1.6)^b^	-8.3 (-17.4 – -2.7)^d^	0.318
*P*	0.098	0.746	
Hip abduction max NDL^a^, OL^c^ [°]	8.8 (1.0 – 13.8)^a^	7.1 (1.8 – 17.4)^c^	0.269
Hip abduction max DL^b^,NOL^d^ [°]	10.9 (7.0 – 19.6)^b^	7.2 (0.1 – 17.5)^d^	**0.002**
*P*	**0.001**	0.575	
Hip abduction rangeNDL^a^,OL^c^ [°]	17.6 (13.5 – 25.5)^a^	15.3 (10.3 – 27.3)^c^	**0.006**
Hip abduction rangeDL^b^NOL^d^ [°]	17.8 (14.7 – 27.8)^b^	16.9 (8.1 – 24.6)^d^	**0.034**
*P*	0.418	0.328	
Knee flexion min NDL^a^, OL^c^ [°]	-2.2 (-9.9 – 3.1)^a^	-3.0 (-7.5 – -0.1)^c^	0.097
Knee flexion min DL^b^, NOL^d^ [°]	-1.4 (-11.8 – 5.2)^b^	-0.5 (-20.4 – 0.1)^d^	0.751
*P*	0.900	0.127	
Knee flexion max NDL^a^, OL^c^ [°]	67.5 (56.3 – 77.7)^a^	58.6 (38.0 – 72.9)^c^	**0.003**
Knee flexion max DL^b^, NOL^d^ [°]	64.7 (57.7 – 76.7)^b^	63.5 (21.6 – 74.7)^d^	0.091
*P*	0.341	0.878	
Knee flexion range NDL^a^, OL^c^ [°]	70.3 (58.7 – 84.3)^a^	63.5 (44.2 – 74.9)^c^	**0.010**
Knee flexion range DL^b^,NOL^d^ [°]	68.1 (61.0 – 74.8)^b^	64.9 (32.8 – 75.7)^d^	0.102
*P*	0.385	0.809	

Data are expressed as medians and 5^th^–95^th^ percentiles

*OL* operated limb in patients^c^, *NDL* Nondominant limb in controls^a^, *NOL* Nonoperated limb in patients^d^, *DL* dominant limb in controls^b^

Bold typeface indicates a statistically significant difference

Data are expressed as medians and 5^th^–95^th^ percentiles. OL: operated limb in patients; NDL: nondominant limb in controls; NOL: nonoperated limb in patients; DL: dominant limb in controls. Bold typeface indicates a statistically significant difference (Table 1).

We observed that the hip flexion range in patients’ NOLs was significantly lower than that in controls’ DLs (*P* = 0.007) (Fig. 2). A comparison between the OL and NOLs (vertical analysis in Table 1) showed no significant differences in the minimum, maximum, or range-of-motion values for hip flexion, hip abduction, or knee flexion in the patient group or between the DLs and NDLs in the healthy controls. Knee flexion differed significantly between the treated patients and the control group, i.e. the OLs and NDLs (maximum, range), but not between the NOLs and DLs (minimum, maximum, range). Furthermore, we observed no significant differences in knee flexion (minimum, maximum, range) between the OLs and NOLs in patients or between the DLs and the NDLs in controls (Table 1).

Differences in ankle dorsiflexion, ankle inversion, and ankle abduction between patients who had undergone Ilizarov therapy and healthy controls are shown in Table 2.

**Table 2:** Differences in ankle dorsiflexion, ankle inversion, and ankle abduction between patients who had undergone Ilizarov therapy and healthy controls

	Control group (*n *= 22)	Patients after surgery (*n* = 23)	*P*
Ankle dorsiflexion min NDL^a^, OL^c^ [°]	-25.0 (-90.8 – -10.5)^a^	-14.3 (-33.5 – -3.5)^c^	** < 0.001**
Ankle dorsiflexion min DL^b^, NOL^d^ [°]	-27.2 (-87.9 – -11.0)^b^	-23.2 (-32.8 – -8.4)^d^	0.075
*P*	0.805	**0.011**	
Ankle dorsiflexion max NDL^a^, OL^c^ [°]	17.7 (5.2 – 62.0)^a^	7.2 (2.2 – 16.8)^c^	** < 0.001**
Ankle dorsiflexion max DL^b^, NOL^d^ [°]	17.7 (10.0 – 54.6)^b^	12.8 (4.5 – 24.4)^d^	**0.003**
*P*	0.991	** < 0.001**	
Ankle dorsiflexion range NDL^a^, OL^c^ [°]	38.2 (14.6 – 146.5)^a^	21.3 (8.6 – 41.8)^c^	** < 0.001**
Ankle dorsiflexion range DL^b^, NOL^d^ [°]	42.6 (27.5 – 142.2)^b^	34.3 (22.4 – 47.1)^d^	**0.004**
*P*	0.511	** < 0.001**	
Ankle inversion min NDL^a^, OL^c^ [°]	- 8.6 (-21.9 – -2.7)^a^	-4.8 (-16.2 – -1.0)^c^	**0.001**
Ankle inversion min DL^b^, NOL^d^ [°]	-6.9 (-44.9 – -0.7)^b^	-7.4 (18.8 – -4.5)^d^	0.982
*P*	0.511	**0.005**	
Ankle inversion max NDL^a^, OL^c^ [°]	12.8 (1.7 – 28.5)^a^	7.2 (0.7 – 15.8) ^c^	** < 0.001**
Ankle inversion max DL^b^, NOL^d^ [°]	15.2 (3.1 – 32.2)^b^	11.9 (1.3 – 33.7)^d^	0.078
*P*	0.722	**0.011**	
Ankle inversion range NDL^a^, OL^c^ [°]	26.7 (14.1 – 38.8)^a^	11.3 (4.3 – 26.9)^c^	** < 0.001**
Ankle inversion range DL^b^, NOL^d^ [°]	22.5 (12.4 – 67.4)^b^	20.7 (7.6 – 42.1)^d^	0.166
*P*	0.751	** < 0.001**	
Ankle abduction min NDL^a^, OL^c^ [°]	-16.9 (-36.6 – -5.8)^a^	-8.0 (-28.4 – -2.4)^c^	** < 0.001**
Ankle abduction min DL^b^, NOL^d^ [°]	-16.9 (-37.3 – -0.7)^b^	-13.2 (-33.1 – -6.0)^d^	**0.025**
*P*	0.707	**0.004**	
Ankle abduction max NDL^a^, OL^c^ [°]	6.5 (-2.9 – 17.0)^a^	4.4 (0.8 – 17.2)^c^	0.231
Ankle abduction max DL^b^, NOL^d^ [°]	10.5 (1.0 – 23.6)^b^	6.8 (2.7 – 13.0)^d^	**0.006**
*P*	**0.036**	0.051	
Ankle abduction range NDL^a^, OL^c^ [°]	24.9 (13.5 – 36.6)^a^	13.9 (4.4 – 32.9)^c^	** < 0.001**
Ankle abduction range DL^b^, NOL^d^ [°]	29.6 (12.7 – 47.4)^b^	19.7 (9.6 – 37.2)^d^	**0.001**
*P*	0.091	**0.003**	

Data are expressed as medians and 5^th^–95^th^ percentiles

*OL* Operated limb in patients^c^, *NDL* Nondominant limb in controls^a^, *NOL* nonoperated limb in patients^d^, *DL* Dominant limb in controls ^b^

Bold typeface indicates a statistically significant difference

**Fig. 3:**
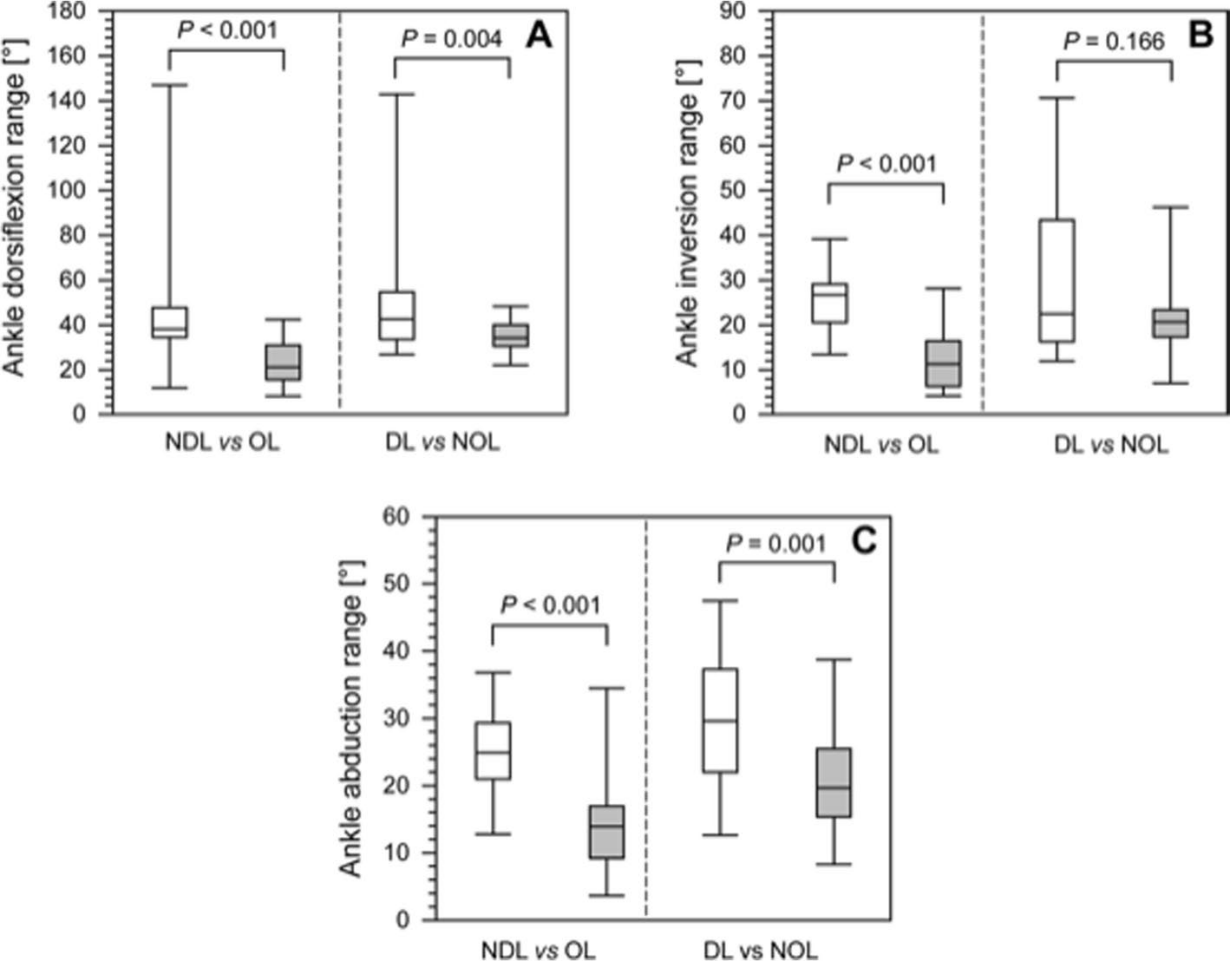
A comparison of the ankle dorsiflexion range (panel **A**), ankle inversion range (panel **B**) and ankle abduction range (panel **C**) for nondominant limbs (NDLs) and operated limbs (OLs) limbs; dominant limbs (DLs) vs nonoperated limbs (NOLs) between healthy controls and patients after treatment with the Ilizarov method. The boundary of the box closest to zero indicates the 25th percentile, a line within the box marks the median, and the boundary of the box farthest from zero indicates the 75th percentile. The whiskers above and below the box indicate the 90th and 10th percentiles respectively. White boxes, healthy people; filled boxes, patients

We observed significant differences between the patients’ OLs and the controls’ NDLs in the ranges of ankle dorsiflexion (*P* < 0.001), ankle inversion (*P* < 0.001), and ankle abduction (*P* < 0.001) (Fig. 3). There were also significant differences in both the ankle dorsiflexion range (*P* = 0.004) and the ankle abduction range (*P* = 0.001) between the patients’ NOLs and the controls’ DLs (Table 2).

We found significant differences between the OL and the NOL in minimum ankle dorsiflexion (*P* = 0.011), maximum ankle dorsiflexion, and the range (*P* < 0.001); in minimum ankle inversion, maximum ankle inversion, and the range of ankle inversion (*P* = 0.005; P 0.011, and *P* < 0.001, respectively); as well as in minimum ankle abduction (*P* = 0.004) and ankle abduction range (*P* = 0.003). We did not find any statistical differences between the DL and NDL in the control group in terms of either minimum or range-of-motion values for ankle dorsiflexion, inversion, or abduction, but we observed significant differences for maximum ankle abduction (*P* = 0.036) (Table 2).

## Discussion

As much as 5–10% of all tibial shaft fractures lead to tibial nonunion [30, 31], which results in a number of complications, including abnormal gait biomechanics, chronic pain, disability, and lower quality of life, additionally generating considerable healthcare costs [32–36]. The prevalence of this injury and the associated dysfunction and complications in various age groups is a serious public health concern.

Nonunion treatment aims to achieve bone union and to restore bone length and the ability to walk. Following treatment, patients are expected to be independent in their everyday functioning, to be able to resume work, and to experience less or no pain. Normal gait and normal functioning require a physiological range of motion [13–20, 37, 38]. Abnormal kinematic parameters may indicate a suboptimal treatment outcome [13–20, 37, 39, 40].

Previous reports demonstrated a high prevalence of range-of-motion limitations, particularly at the ankle and knee, in patients with tibial nonunion [1, 3, 4, 6, 8, 9]. This is consistent with our observations that post-treatment range-of-motion assessment is important from the point of view of patients, orthopedists, and physiotherapists because it helps identify joints with a limited range of motion, which thus require more intense rehabilitation procedures and exercises. Before the surgery, it is important for patients to know which ranges of motion in particular joints will return to normal and which ranges of motion will still be limited after treatment. However, there have been no measurements to date of the range of motion in lower limb joints during ambulation. The range of motion has been conventionally measured in specific positions allowing the measurement of the full range of motion at a given joint. Conducting measurements during walking helps assess the effects of treatment and the range of motion in the patients’ joints under weight-bearing conditions.

By comparing hip flexion in the patients’ OLs with that in the controls’ NOLs our study demonstrated significantly greater values in the control group, which indicates that, 24–48 months after their surgery, the patients had worse gait function than the healthy controls. Nevertheless, interestingly, the patient group was also disadvantaged in terms of minimum hip flexion and hip flexion range in the NOL, which indicates an effect of tibial nonunion on the function of the healthy limb.

Bilateral hip joint movement limitations observed in the assessed patients may be a result of long-term restrictions in physical activity and movement as well as instability of the affected lower leg, which could lead to secondary partial muscle atrophy of both lower limbs and degenerative changes in the hip joint. Also, the relatively long-lasting pathology of the musculoskeletal system in the form of a tibial nonunion could have influenced the development of compensatory gait mechanisms with a limited range of motion in both hips. According to our study in the group of healthy people and similarly in the group of patients there was no difference between hip range of motion in the DL and in the NDL in controls and hip range of motion in the OL and NOL in patients. However, we observed similar values of hip range of motion in both limbs (i.e. in the OL and in the NOL) in patients, which differed significantly from those in healthy controls.

Many studies have assessed goniometer-measured ankle and knee ranges of motion at rest in patients who had undergone osteotomy and deformity correction using the Ilizarov method [21–23]. Conversely, studies assessing gait parameters following Ilizarov treatment did not evaluate ranges of motion [16, 17]. Earlier measurements of lower limb kinematic parameters following treatment of a musculoskeletal disorder were conducted using optical measurement systems and the use of cameras [18, 19]. This limited the accuracy of range-of-motion assessments [39]. The Noraxon MyoMOTION System used in our study ensures very accurate, reproducible, and objective records of joint mobility [24–27]. The figures on joint range of motion obtained in our study are consistent with those measured in previous studies on the range of motion in healthy individuals [41–43].

Other authors compared preoperative and postoperative ranges of motion at the ankle and knee joints in patients treated with tibial osteotomy. Rozbruch et al. demonstrated a lack of significant differences between the preoperative and postoperative ranges of motion at the ankle and knee joints in patients treated with tibial osteotomy with an external fixator [21]. The postoperative mean knee extension, knee flexion, and ankle dorsiflexion angles measured in 102 patients were 0°, 125°, and 11°, respectively [21].

Osman et al. achieved ankle dorsiflexion in the 0–20° range following the use of the Ilizarov method in pilon fractures [22]. These ankle dorsiflexion values were similar to these achieved in our study (21.3 ). Wang, who assessed fourteen patients who had undergone equinovarus foot deformity correction using the Ilizarov method, reported a mean ankle dorsiflexion of 8.3° [23]. The differences in joint range of motion values between our patient group and the literature data [21–23] may be due to the greater severity of injuries and deformities of the lower limbs in our tibial nonunion group compared with those in the patients assessed by other authors.

In the case of the ankle, we assessed dorsiflexion, inversion, and abduction, and the post-treatment measurements in the operated limbs differed significantly from the measurements in the control group for all the examined parameters. This indicates that, despite treatment, ankle joint mobility is not as good as in healthy individuals.

The comparison of the NOLs from the patient group with the DL in the control group additionally demonstrated that hip abduction, hip flexion, ankle dorsiflexion. and ankle abduction are also statistically significantly lower in the patient group. We consider this to be an interesting observation that requires further research on more people. The limitations in NOL mobility may result from the fact that the analyzed patients, due to tibia nonunion, had for a long time limited mobility, daily physical activity, and recreational physical activity.

The Noraxon MyoMOTION System has been used to assess joint mobility in various sports disciplines (including running) as well as lower limb disfunction other than tibial nonunion [26].

Manjar et al. reported reduced ankle dorsiflexion following tibia fracture treatment with an external fixator [19]. Madhusudhab observed limited ranges of motion at the ankle and knee in all patients after tibial nonunion treatment using the Ilizarov method [8]. Sanders reported limited ranges of motion in 15.8% of patients who had undergone treatment for tibial nonunion using the Ilizarov method [9]. The ranges of motion at the knee and hip in our patients were consistent with those reported by other authors [21–23].

In our study group only the knee flexion and ankle inversion ranges in the patients’ NOLs did not significantly differ from those in the controls’ DLs. However, the other kinematic parameters evaluated in the patient group were significantly lower than those in the control group. This may have been due to several factors, including a too short postoperative rehabilitation period, which resulted in limitations in the range of motion. Our physiotherapeutic management protocol included at least four sessions a week during the first six to eight weeks after surgery. During this period, the physiotherapeutic efforts focused mainly on managing pain severity while improving the range of motion, muscle strength, and teaching the patients normal locomotion. Subsequently, patients received a special individualized rehabilitation regimen for use at home and, once a week, under a physiotherapist’s supervision. We believe that physiotherapeutic supervision and intensive rehabilitation should last considerably longer, until treatment is complete.

Another undesirable effect of treatment observed in some patients were extensive scars and adhesions involving the subcutaneous tissue, muscles, and tendons. The Achilles tendon plays an important role in the movement at the knee [26]. Moreover, traumatic and degenerative joint changes and pain may contribute to range-of-motion limitations [38, 40]. The observed range-of-motion differences may have also been due to compensatory mechanisms [24]. A change in the range of motion at one joint results in compensatory changes in the range of motion of other joints [24]. Moreover, increasing gait speed increases the joint range of motion [26]. Another factor contributing to joint stiffness may have been the long immobilization with an external fixator [19].

### Limitations

One of the limitations of our study is its retrospective character, which was due to the impossibility of assessing kinematic parameters in patients prior to surgery, since they were either unable to walk or their walking ability was considerably impaired, due to pain and pathological mobility at the tibial nonunion site. Nonetheless, range-of-motion studies carried out by other authors were also retrospective [19, 22, 23]. Due to the small number of patients after tibial nonunion treatment, it was not possible to select a uniform group of patients in terms of the number of previous surgeries and the exact site of tibial nonunion (in the same sections of the bone). Another limitation was the small sample size; however, most other authors who assessed kinematic parameters also used study groups of similar or smaller sizes [19, 22–27], as it is difficult to accrue a large population of patients who consent to undergo additional evaluations. Moreover, we had no data on either patients’ or volunteers’ unhealthy habits, such as systematic smoking, unhealthy diets [44], or comorbid metabolic conditions, which may potentially affect functional recovery, based on laboratory test results such as fasting blood glucose or insulin levels; instead, the only type of comorbidity-associated data we collected was limited to that elicited at history-taking. Our study volunteers were deemed healthy based on a general history-taking negative for metabolic conditions, including diabetes. However, individual lifestyle choices, such as smoking, were considered in the analysis, since we assumed that such parameters have no impact on kinematic gait parameters in the control group.

Another limitation of our work is the lack of assessing the residual pain and its correlation with range of motion; however, other authors also did not assess the correlation between residual pain and range of motion [21–23, 37].

The strengths of our study include the uniform postoperative management and rehabilitation regimen, the long follow-up, the carefully selected control group, and the reproducible assessment of kinematic parameters using the objective and accurate Noraxon MyoMOTION System [24–27].

The statistically significant differences between the OLs and NOLs in patients demonstrated by the measurement method employed in our study are significant from the clinical point of view and indicate that the accuracy of measurements may help optimize and personalize treatment and rehabilitation for subsequent patients treated with the Ilizarov method.

Our retrospective study assessed kinematic parameters after treatment. The observed range-of-motion abnormalities may have been a product of the initial injury that led to tibial nonunion and other surgical procedures that the patients underwent before the Ilizarov treatment. Abnormal joint mobility may also have been due to the Ilizarov treatment itself.

## Conclusion

Tibial nonunion treatment using the Ilizarov method does not ensure complete normalization of kinematic parameters assessed 24–48 months following the completion of treatment and rehabilitation.

The kinematic parameter values in the NOLs of patients after Ilizarov treatment and in the DLs of healthy individuals.

## Data Availability

The datasets used and/or analyzed during the current study are available from the corresponding author on reasonable request. The data are not publicly available due to privacy.
